# Fenfluramine for seizures in Dravet syndrome and Lennox-Gastaut syndrome: Mechanisms, clinical evidence, safety, and practical integration

**DOI:** 10.1016/j.ebr.2026.100876

**Published:** 2026-06-04

**Authors:** József Janszky, József Janszky, Réka Horváth

**Affiliations:** a Department of Psychiatry and Psychotherapy, Medical School, Clinical Centre, University of Pécs, Rét u. 2, 7623 Pécs, Hungary; b Department of Neurology, Medical School, Clinical Centre, University of Pécs, Rét u. 2, 7623 Pécs, Hungary

**Keywords:** fenfluramine, Dravet syndrome, Lennox-Gastaut syndrome, sigma-1 receptor, serotonin, echocardiography, antiseizure medication

## Abstract

Developmental and epileptic encephalopathies such as Dravet syndrome and Lennox–Gastaut syndrome remain highly drug resistant and are associated with substantial neurodevelopmental, behavioral, and caregiver burdens. Fenfluramine, repurposed at low antiseizure doses, is approved as adjunctive therapy for both syndromes and has a distinct multimodal mechanism that combines serotonergic modulation with positive allosteric activity at the sigma-1 receptor. This narrative review summarizes key translational insights and the pivotal clinical trial evidence in Dravet syndrome and Lennox–Gastaut syndrome, including long-term open-label extension and emerging real-world data. Across randomized studies, fenfluramine reduces convulsive seizures in Dravet syndrome and drop seizures in Lennox–Gastaut syndrome, with clinically meaningful responder rates and durability over time. Particular attention is given to neurocognitive and behavioral outcomes, as caregiver-reported improvements in executive function have been observed in Dravet cohorts and may not be fully explained by seizure reduction alone. Given historical associations between high-dose fenfluramine and cardiopulmonary adverse effects, we also review cardiovascular safety findings at antiseizure doses and the rationale for baseline and periodic echocardiographic monitoring. Finally, practical dosing, drug–drug interaction considerations, and an integration algorithm are provided to support routine clinical implementation across pediatric and adult care pathways.

## Highlights

• Fenfluramine has randomized-trial evidence for reducing convulsive seizures in Dravet syndrome and drop seizures in Lennox-Gastaut syndrome.
• Emerging caregiver-reported data suggest improvements in everyday executive function in both Dravet syndrome and Lennox-Gastaut syndrome, although objective prospective confirmation is still needed.
• Practical use depends on dose caps, stiripentol status, CYP inhibitor/inducer interactions, appetite/weight monitoring, and scheduled echocardiography.

## 1 Introduction

Developmental and epileptic encephalopathies (DEEs) remain largely drug-resistant and are associated not only with a high seizure burden, but also with persistent neurocognitive and behavioral comorbidities, impaired quality of life, and caregiver burden. Within this spectrum, Dravet syndrome (DS) and Lennox–Gastaut syndrome (LGS) are phenotypically and etiologically distinct, but they share early onset, multiple seizure types, the need for polytherapy, and adverse effects on clinical outcomes, including cognitive development and SUDEP risk [1].

Current standards (e.g., valproate, clobazam, ketogenic diet, vagus nerve stimulation) often provide insufficient seizure control, creating a need for well-integrated adjunctive therapies with different mechanisms of action that are evidence-based in reducing convulsive seizures and drop attacks and have a manageable safety profile. Low-dose fenfluramine (FFA), which has come to the fore through drug repositioning, has emerged as a separate therapeutic option and is approved in several regions, including the EU and the US, as an adjunctive therapy for DS and LGS from the age of 2 years [2], [3]. The use of the drug is subject to a strict cardiovascular monitoring program that requires baseline and periodic echocardiography due to the risks associated with historical anorectic doses. It is important to note that in epilepsy programs, valvulopathy or pulmonary arterial hypertension (PAH) has not been reported in the antiepileptic dose range, while label-based monitoring is consistently enforced [2], [3]. Mechanistically, FFA has a dual effect: on the one hand, it enhances serotonergic transmission and activates certain 5-HT receptors (particularly 5-HT1D/2C, presumably 5-HT4), and on the other hand, it acts as a positive allosteric modulator on the sigma-1 receptor—this combination gives it a unique pharmacological profile among current antiepileptic drugs [4], [5], [6]. Translational and clinical data also indicate that, in addition to reducing seizures, FFA may affect everyday executive functions (EF), especially in younger DS populations, and that this improvement can only be partially explained by the reduction in seizure burden [7], [8]. Based on these findings, a review integrating the mechanistic basis with the results of key studies in DS and LGS, long-term follow-up and real-world data, and cardiovascular surveillance requirements is warranted. This summary also proposes practical, integrable algorithms for the clinical integration of FFA [2], [3], [4].

The added value of this review is a clinically practical synthesis that links mechanism, randomized and extension evidence, executive-function findings, emerging data beyond DS/LGS, dose caps, and drug-drug interaction management into a phenotype-guided integration strategy for routine care.

## 2 Pharmacology and mechanisms

FFA increases extracellular serotonin levels and activates a specific subset of 5-HT receptors, while its active metabolite, norfenfluramine, contributes to the overall pharmacology (particularly 5-HT1D/2C and probable 5-HT4 involvement in cognitive function) [4], [5], [6]. In preclinical systems, including SCN1A mutant zebrafish, pharmacological analysis consistently points to 5-HT1D/2C-mediated seizure suppression [4], [9], [10], [11]. This serotonergic pathway is complemented by a second, mechanistically distinct axis: fenfluramine acts as a positive allosteric modulator on sigma-1 receptors (σ1-R), a chaperone-like protein that is abundant in endoplasmic reticulum (ER) membranes associated with mitochondria. σ1-R modulation stabilizes intracellular Ca^2+^ handling and stress response signaling and influences network-level excitability — properties that form a biologically plausible bridge between seizure control and seizure-free outcomes such as improvements in executive function (EF) observed in clinical cohorts [4], [5], [6].

At the stereochemical level, converging evidence suggests that both racemic FFA and (+)-norfenfluramine have anticonvulsant effects, while enantiomer-sensitive differences in efficacy and tolerability are apparent in model systems. In zebrafish Dravet paradigms, experiments with enantiomers show that norfenfluramine is not merely a passive metabolite but an active contributor with distinct chiral preferences in readouts [12]. Data from stereoselective mice complement these observations and suggest pharmacodynamic nuances that could be exploited in future optimization strategies (e.g., chiral weighting or metabolite-aware dosing windows) [13]. Together, these results support a multicomponent mechanism in which (i) presynaptic 5-HT release and selective receptor activation reduce pathological synchronization, (ii) σ1-R-related modulation refines cellular stress and calcium signaling, and (iii) the specific contribution of enantiomers fine-tunes efficacy/tolerability profiles in circuits [4], [5], [6], [12], [13].

Beyond receptor pharmacology, characterization of the in vitro reaction phenotype clarifies why fenfluramine behaves primarily as a drug-drug interaction (DDI) victim rather than a broad perpetrator. Norfenfluramine formation is driven primarily by CYP2D6, CYP2B6, and CYP1A2, and to a lesser extent by CYP2C9, CYP2C19, and CYP3A4/5, implying susceptibility to potent inhibitors/inducers and genetic polymorphisms at these loci [14]. Transporter studies also show that fenfluramine/norfenfluramine do not constitute substrates for key renal/hepatic transporters (BCRP, OAT1/3, OCT2, MATE1/2-K) [14]. In practice, this profile is consistent with the label guidance: upper dose limits and modifications focus more on concomitant use of CYP inhibitors and treatments containing stiripentol than on transporter-mediated risks [2], [3], [14].

5-HT1D/2C-mediated inhibition may reduce thalamocortical hypersynchrony and photosensitivity (consistent with the responses of photosensitive phenotypes), while σ1-R modulation may stabilize interneuron-pyramidal cell connectivity under cellular stress, preserving oscillatory balance in frontal-temporal circuits implicated in DS and LGS [4], [5], [6], [9], [10], [11].

This dual model provides a simple explanation for (a) the significant reduction in seizures (b) patient retention and sustained response in long-term open-label extension (OLE) cohorts (c) the improvement in executive function (EF) reported by caregivers, which can only be partially explained by changes in seizure frequency—i.e., a probable mechanism-function relationship consistent with translational evidence [4], [5], [6], [7], [8].

### 2.1 Clinical efficacy in Dravet syndrome

In two randomized, double-blind, placebo-controlled phase 3 studies, FFA (doses up to 0.7 mg/kg/day; lower maximum dose when combined with stiripentol) was associated with a significant reduction in the frequency of monthly seizures [15], [16]. In both studies, the effect of treatment was observed early in the dose titration phase and was maintained during the maintenance phase, with a clear dose-response pattern between the predetermined doses [15], [16].

The response rate was consistently higher than placebo for conventional thresholds (≥50% and ≥ 75% reduction in monthly seizures), and the beneficial effects extended to clinically meaningful secondary outcomes, including overall improvement reported by caregivers and physicians [15], [16]. Time-to-event analysis confirmed the magnitude of the effect: measures such as the longest seizure-free interval after baseline and time to first seizure favored FFA (e.g., median longest seizure-free interval ∼ 25–30 days vs. ∼10–15 days for placebo), indicating not only fewer events but also longer intervals between seizures [2], [5], [15], [16]. The efficacy results were consistent in clinically relevant subgroups (e.g.,polytherapy, treatments containing stiripentol), supporting generalizability to DS practice [15], [16].

Open-label extension data confirm these results, showing sustained efficacy over several years, with maintained response rates, stable dosing within the labeled limits, and favorable benefit-risk profiles with protocol-specific echocardiographic monitoring [17]. Extended cohorts also suggest additional clinical benefits consistent with real-world priorities, such as an increase in seizure-free days, a decrease in reliance on rescue medications, and a decrease in the number of urgent medical visits among responders, all while maintaining mandatory cardiological safety monitoring [2], [17]. Real-world evidence from national and multicenter programs is consistent with the study description: long-term use is associated with seizure control, high treatment retention, and measurable reductions in healthcare resource utilization (e.g., emergency care, hospitalizations, condition-related care) within a structured monitoring framework [18]. Overall, the DS evidence suggests that FFA results in robust and sustained seizure reduction, improves clinically meaningful time-based outcomes. [2], [5], [15], [16], [17], [18].

### 2.2 Clinical efficacy in Lennox-Gastaut syndrome

In a randomized, double-blind study involving 263 patients, a dose of 0.7 mg/kg/day of FFA resulted in a statistically significant reduction in seizure frequency compared to placebo, with the effect of treatment occurring during dose adjustment and persisting during maintenance treatment [19]. Antiseizure effect is effective in all types of seizures of LGS “drop”seizures (primarily atonic, tonic, and tonic-atonic seizures), the reduction was in favor of FFA, consistent with clinically relevant endpoints of the syndrome that measure fall-related morbidity [19]. Exploratory subgroup analyses showed a marked effect in patients with generalized tonic-clonic seizures (GTC), who typically have higher overall morbidity [19].

Noteworthy is the consistency with regulatory summaries: the US labeling describes a rightward shift in the distribution of responders, with a higher proportion of patients achieving at least a 50% reduction in epileptic drop attacks with FFA than with placebo, accompanied by clinically reported outstanding global improvement [3]. These categorical improvements are consistent with practical endpoints assessed by caregivers and clinicians (injury-risk falls, need for rescue medication, and overall functional stability. In an open-label extension of the randomized trial, sustained reductions were observed over approximately 12–15 months for both “drop’” and” non-drop” seizure types, and convergent impressions reported by caregivers and clinicians supported the durability and functional benefits [20]. Treatment retention was high, dosing remained within the labeled range, and safety, including mandatory echocardiographic monitoring, was consistent with the double-blind phase, indicating that efficacy is not associated with the emergence of cardiopulmonary risk signs when monitoring protocols are adhered to [20].

Overall, based on the LGS evidence, FFA can be considered a clinically significant, durable adjunctive therapy that favorably influences responder categories, improves overall impressions, and is compatible with standard polytherapy in this highly refractory population [3], [19], [20].

A recent final open-label extension analysis further supports the long-term impact of fenfluramine on patients and caregivers in LGS, while maintaining the same need for structured safety monitoring [21].

### 2.3 Other developmental and atypical epilepsies: CDKL5 deficiency disorder and photosensitive phenotypes

Fenfluramine has also been explored in CDKL5 deficiency disorder (CDD), although this remains outside the currently approved DS/LGS indications. In an early open-label series of six patients with highly refractory CDD, fenfluramine was used at 0.4 mg/kg/day or 0.7 mg/kg/day, up to a maximum of 26 mg/day. Among five patients with tonic-clonic seizures, treatment was associated with a median 90% reduction in seizure frequency, while tonic seizures decreased by 50–60% in two patients. No valvular heart disease or pulmonary arterial hypertension was reported, although the sample size and uncontrolled design preclude firm conclusions. More recently, the phase 3 GEMZ trial in CDD reported statistically significant reductions in countable motor seizure frequency with fenfluramine 0.7 mg/kg/day, maximum 26 mg/day, compared with placebo, with no new safety signals; however, these results should be interpreted cautiously until full peer-reviewed publication and regulatory evaluation are complete [22], [23].

Sunflower syndrome—a self-induced photosensitive epilepsy characterized by repetitive hand movements toward light—prospective, open observations suggest that the use of supplemental FFA results in a clinically significant reduction in the daily frequency and duration of the typical seizure, while no echocardiographic abnormalities were observed during short-term follow-up under label-prescribed supervision [12], [24]. In these small cohorts, improvement was recorded in structured caregiver logs and video-documented counts, and was accompanied by better tolerability (adverse events typical of the broader program, e.g., appetite/weight effects) and stable dosing within the approved range; Importantly, mandatory ECGs were unchanged at baseline and at the end of the study, and there were no signs of valvulopathy or pulmonary arterial hypertension during the observation period [12], [24]. Although the study design was uncontrolled, the direction and internal consistency of the results are consistent with a mechanistic explanation: 5-HT1D/2C serotonergic binding plays a role in reducing thalamo-cortical hypersynchrony and photoparoxysmal susceptibility, while σ₁ receptor modulation provides a likely cellular link (ER–mitochondrial Ca^2+^ handling, stress response signaling) to stabilize hyper-excited networks under light exposure [4], [5], [6], [9], [10], [11], [12], [24].

Clinically, the evidence seems to go beyond the seizure with typical hand movements, and in some participants, non-hand movement events (e.g., absence or myoclonic components sometimes accompanying Sunflower syndrome) show a decreasing trend. Important limitations remain—small sample sizes, lack of randomized comparators, short follow-up, and potential expectation bias—and therefore these data should be considered preliminary, and confirmatory RCTs are warranted.

### 2.4 Neurocognitive and behavioral outcomes

Post-hoc and targeted analyses using the Behavioral Rating Inventory of Executive Functions (BRIEF/BRIEF-P) consistently show clinically significant improvements in everyday executive functions (EF) in children with DS receiving supplemental FFA treatment, including preschoolers [7], [8]. Multiple analytical approaches suggest that these EF improvements are only partially attributable to a reduction in seizures [7], [8]. Consistent with this, exposure-response patterns can be observed in preschool children: higher, label-matched doses were associated with greater EF improvement, and some children showed EF improvement despite not reaching the conventional ≥50% seizure response threshold [4], [5], [6], [7], [8], [9]. Overall, these results are consistent with the mechanistic likelihood that a serotonergic spine (namely 5-HT1D/2C, likely with 5-HT4 relevance in cognitive function) coupled with sigma-1 receptor positive allosteric modulation, which may stabilize intracellular calcium handling and stress response signaling in networks involved in behavioral control and metacognition [4], [5], [6], [9].

Emerging data also extend the executive-function signal to Lennox-Gastaut syndrome. Post hoc analyses from the phase 3 LGS program evaluated caregiver-rated everyday executive function using BRIEF-based instruments. In adults with LGS, BRIEF-A analyses showed improvement or stabilization in the Behavioral Regulation Index, Metacognition Index, and Global Executive Composite in fenfluramine-treated groups, whereas placebo-treated patients tended to worsen. In the randomized phase and open-label extension, the proportion of patients with clinically elevated T-scores decreased after fenfluramine exposure, and correlations between changes in fall-associated seizures and changes in BRIEF-A scores were negligible to weak. These findings suggest that caregiver-perceived executive-function benefits in LGS may not be fully explained by seizure reduction alone. However, because these analyses are post hoc and rely on caregiver-rated rather than performance-based cognitive outcomes, they should be interpreted as hypothesis-generating and should be confirmed prospectively [25], [26], [27].

Methodologically, studies use EF measures reported by caregivers, potential confounder (sedation/drowsiness, appetite/weight change, sleep consolidation) and background polytherapy (e.g., clobazam, stiripentol, cannabidiol)—require careful sensitivity analyses to distinguish true improvements in executive function from indirect effects of better seizure control or side effect profiles.

### 2.5 Safety and tolerability

Cardiovascular safety: In key DS/LGS studies and their long-term extensions conducted at antiseizure doses, no cases of valvulopathy or pulmonary arterial hypertension were observed during protocol-mandated echocardiographic monitoring. However, post-marketing experience has identified such cases, and therefore the continuous monitoring required by the label remains essential [2], [3]. Both EU and US labeling require a structured ECHO program—a baseline examination, followed by examinations every 6 months for the first 2 years and annually thereafter; in the US, a final ECHO examination is also required 3–6 months after discontinuation of treatment [2], [3]. In practice, each assessment should document valve morphology and function (e.g., mitral, aortic, and tricuspid valve leaflet thickening, motion restriction, and degree of regurgitation) as well as noninvasive estimation of pulmonary pressure with clinical correlation (new exertional dyspnea, edema, murmur changes). If the initial ECHO result is inconclusive or shows pre-existing valve abnormalities, treatment should be postponed until the cardiologist's opinion is obtained, and in the event of any new or progressive valve changes during treatment, the multidisciplinary team should re-evaluate and repeat the benefit-risk discussion [2], [3]. These safety measures reflect the risks of valvulopathy or pulmonary arterial hypertension experienced with high-dose appetite suppressant combinations in the 1990s [28], [29], [30].

Other side effects and interactions – The most common events during treatment are: decreased appetite, weight loss, diarrhea, fatigue, and drowsiness. Because weight-based dosing and effects on appetite overlap, routine monitoring of growth/BMI and early nutritional counseling are practical adjuncts to treatment. Daytime sleepiness/sedation should be distinguished from improved nighttime sleep and managed by dosing timing or gradual titration. The maximum daily dose is limited in treatments containing stiripentol, and dose modification is recommended according to the label recommendation in cases of strong CYP1A2/2D6 inhibitors and severe renal/hepatic impairment [3]. Due to the serotonergic pharmacology of fenfluramine, physicians should review the concomitant use of serotonergic agents (e.g., SSRIs/SNRIs, triptans) and avoid agents that significantly increase serotonergic tone (e.g., MAOIs) due to the theoretical risk of serotonin toxicity; if new behavioral or autonomic symptoms appear, a thorough medical history and coordination of medication are essential [3]. Finally, although transporter-mediated interactions are not expected to be dominant at therapeutic concentrations, the complex polytherapy of DS/LGS warrants of drug-drug interaction (DDI) and consultation with the pharmacy, especially when adding or discontinuing enzyme inhibitors/inducers [3]. In summary, a structured plan—scheduled monitoring of ECHO, monitoring of body weight/BMI, checking for concomitant medications [2], [3], [28], [29], [30].

Strong enzyme inducers should be considered separately from CYP inhibitors. Fenfluramine is metabolized mainly through CYP1A2, CYP2B6, and CYP2D6 pathways; therefore, strong CYP1A2 or CYP2B6 induction may lower fenfluramine exposure and reduce efficacy. According to the EMA product information, if co-administration with a strong CYP1A2 or CYP2B6 inducer is unavoidable, patients should be monitored for reduced efficacy and a fenfluramine dose increase may be considered, provided that it does not exceed twice the maximum daily dose, i.e., 52 mg/day. If the inducer is discontinued during maintenance treatment, gradual reduction back to the pre-inducer fenfluramine dose should be considered. The US prescribing information is more conservative and recommends avoiding strong CYP1A2, CYP2B6, or CYP3A inducers where possible; if co-administration is necessary, patients should be monitored for reduced efficacy and dose adjustment should follow the local label. Thus, for international practice, inducer management should be explicitly region-specific and based on the applicable SmPC/prescribing information [2], [3], [14].

### 2.6 Practical use: dosing and monitoring

Dosing should be expressed explicitly as both per-dose and total daily dose to avoid ambiguity. Treatment is initiated at 0.1 mg/kg twice daily, i.e., 0.2 mg/kg/day. In patients not receiving stiripentol, the dose may be titrated to 0.35 mg/kg twice daily, i.e., 0.7 mg/kg/day, with a maximum total daily dose of 26 mg/day. In patients receiving concomitant stiripentol plus clobazam, the recommended maintenance dose is 0.2 mg/kg twice daily, i.e., 0.4 mg/kg/day, with a maximum total daily dose of 17 mg/day. When using strong CYP1A2/2D6 inhibitors concomitantly (or in cases of severe renal impairment), limit the total daily dose to 20 mg/day for treatments without stiripentol and maintain 17 mg/day for treatments containing stiripentol; in cases of hepatic impairment, follow the instructions on the label (Table 2) [3]. When discontinuing treatment, always reduce the dose gradually to reduce the risk of recurrent seizures/status epilepticus [2], [3].

Fenfluramine oral solution (2.2 mg/mL) — dose & volume calculation (BID) Use a graduated oral syringe (not a household spoon). Volume per dose (mL) = target mg per dose ÷ 2.2 mg/mL; round to the nearest 0.1 mL. Daily caps: ≤26 mg/day (without stiripentol) and ≤ 17 mg/day (with stiripentol).

Administer twice daily at fixed times; minor adjustments to dosing times (earlier evening dose) may help manage somnolence without compromising exposure [3].

When adding stiripentol, reduce the FFA to the lower maximum value and readjust for effect/tolerability; when withdrawing stiripentol, re-evaluate upwards toward the maximum value without stiripentol on the label, increasing in weekly increments. When starting or stopping treatment with strong CYP1A2/2D6 inhibitors, the upper dose limit and clinical effect should be reevaluated (exposure may change significantly) [3]. Since fenfluramine primarily acts as a DDI victim (multipathway CYP biotransformation) rather than a transporter-mediated perpetrator, the interaction focuses on enzyme inhibitors/inducers rather than renal/hepatic transporters [14]. Avoid MAO inhibitors and use caution with other serotonergic agents (additive risk) [3], [14].

At each visit, document weight/BMI (due to effects on appetite/weight), vital signs, adverse events (drowsiness, fatigue, GI), and changes in concomitant medications (new SSRIs/SNRIs, triptans, inhibitors/inducers). If the degree of valve regurgitation or pulmonary artery systolic pressure (PASP) changes significantly, bring forward the ECHO and discuss the benefits and risks with the cardiologist before continuing [2], [3].

FFA is compatible with ketogenic diets and feeding tubes. For school-aged patients, consult with caregivers about dosing timing around classes/meals; for underweight or rapidly growing children, re-evaluate mg/kg monthly to avoid accidental underdosing due to weight changes.

### 2.7 Positioning among therapies

In DS, FFA complements stiripentol and cannabidiol/clobazam as evidence-based adjunctive therapy and can be easily integrated into existing polytherapy regimens. Comparative and network meta-analyses show that, for conventional response thresholds (≥50% and ≥ 75% reduction in seizures), FFA performs in the same range of efficacy as first-line adjunctive drugs, such as stiripentol, and generally compares favorably with cannabidiol-based treatments [1], [31]. These analyses, together with extended/real-world retention data, support the earlier introduction of FFA in DS when the generalized tonic-clonic seizure frequency is high or when previous adjunctive medications have resulted in only a partial response, although true, direct comparative evidence is still lacking [1], [31].

In LGS, FFA complements rufinamide, clobazam, lamotrigine, topiramate, and cannabidiol, with randomized data showing clinically significant reductions in seizure frequency and extension results suggesting broader seizure type improvement during continuous treatment [19], [20].

No head-to-head trial has defined a single “best” fenfluramine combination. In practice, combination selection should be phenotype- and tolerability-guided. In Dravet syndrome, fenfluramine is most often considered within valproate-, clobazam-, and/or stiripentol-based regimens, or as an alternative/add-on when prior adjunctive therapy provides only partial benefit. In Lennox-Gastaut syndrome, it may be particularly useful when drop seizures or generalized tonic-clonic seizures remain prominent despite standard adjunctive options. Once a clinically meaningful response is achieved, simplification of background therapy may be considered to reduce sedation, gastrointestinal burden, or appetite/weight effects, but only gradually and with seizure-diary monitoring.

Dose capping is clinically relevant, especially in heavier adolescents and adults. The 26 mg/day cap without stiripentol corresponds to the full 0.7 mg/kg/day target only up to approximately 37 kg; above this weight, the delivered mg/kg exposure is progressively lower. Similarly, the 17 mg/day cap used with stiripentol-containing regimens corresponds to 0.4 mg/kg/day only up to approximately 42.5 kg. Therefore, apparent partial response in heavier patients should prompt review of actual mg/kg exposure, adherence, interacting co-medications, and seizure phenotype before concluding inefficacy [2], [3].

## 3 Conclusions

Overall, evidence from two phase 3 trials in DS and one trial in LGS—reinforced by long-term extensions and convergent real-world cohorts—supports fenfluramine as an effective and durable adjunctive treatment for these two typical developmental and epileptic encephalopathies. The benefits are not limited to a reduction in the mean frequency of seizures: changes in the proportion of categorical responders (≥50% and ≥ 75%) and time-based measures (longer seizure-free intervals, more seizure-free days) suggest clinically significant benefits that are important to both families and services. The safety of clinical programs is reassuring with regard to PAH/VHD signs, and the existence of post-marketing cases justifies the echocardiographic monitoring is required, resulting in a favorable benefit-risk ratio when dose limits (particularly for stiripentol) and CYP1A2/2D6 inhibitor restrictions are adhered to.

Mechanistically, dual pharmacology—serotonergic effects (5-HT1D/2C, with probable 5-HT4 relevance) and sigma-1 receptor modulation—provides a biologically coherent explanation for the broad seizure control and provides a hypothesis-generating link to the improvement in daily executive functions reported by caregivers, which appears to be only partially a consequence of seizure reduction. These neurocognitive signals are clinically relevant, particularly in preschool-aged DS, but now require prospective, performance-based confirmation to move from promising observation to standard expectation.

In terms of treatment sequence, network-level comparisons suggest that FFA is among the first-line adjunctive agents for DS alongside stiripentol and often compares favorably to cannabidiol-based treatments in terms of traditional response endpoints, while in LGS it results in a reduction in seizure frequency and broader seizure type benefits in extended settings. The lack of direct comparative studies argues for disciplined, phenotype-driven use: priority should be given to patients with high GTC or drop seizures, reliable monitoring of ECHO checkpoints should be ensured, and therapy should be embedded in a coherent polytherapy rather than completely replacing treatment.

In practice, success depends on algorithmic implementation (Table 1.): weight-based titration to the maximum values indicated on the label (lower with stiripentol), DDI vigilance focusing on CYP inhibitors/inducers (fenfluramine as a DDI “victim”) and scheduled echocardiography with low thresholds for cardiac evaluation if baseline or interval results are atypical. Real-world data suggest reduced healthcare utilization and high retention rates, consistent with the pharmacology of the drug, its demonstrated efficacy in clinical trials, and service outcomes.

**Table 1:** Recommended algorithm for patients with Dravet syndrome or Lennox-Gastaut syndrome (childhood and adulthood).

#	Step	What to do	Key parameters / thresholds	Ref.
1	Confirm indication & baseline risk	Verify DS/LGS with persistent seizures despite optimized SOC; capture cardiac baseline.	Baseline transthoracic ECHO: valve morphology, regurgitation grade, estimated PASP; review cardiac history/risk factors.	[2], [3]
2	Screen contraindications & counsel	Check for drug/condition contraindications; align family expectations and monitoring duties.	Avoid MAOIs; review serotonergic agents (SSRIs/SNRIs, triptans); explain US REMS / EU CAP, growth/BMI tracking, and expected timelines.	[2], [3]
3	Start & titrate (BID)	Initiate low; up-titrate to labeled ceilings by tolerability/response.	Start 0.1–0.2 mg/kg/day (split BID). No stiripentol: target 0.35 mg/kg BID (0.7 mg/kg/day), max 26 mg/day. With stiripentol+clobazam: 0.2 mg/kg BID (0.4 mg/kg/day), max 17 mg/day. Increase stepwise (e.g., weekly).	[3]
4	Administration	Calculate per-dose volume and choose tools that improve accuracy and tolerability.	Oral solution 2.2 mg/mL; per-dose volume = (mg per dose) ÷ 2.2; round to nearest 0.1 mL; use graduated oral syringes; consider evening dose timing to mitigate somnolence.	[2], [3]
5	Check co-medications at each change	Reconcile meds; constrain dose under interacting conditions.	Strong CYP1A2/CYP2B6 inducers (EU) and strong CYP1A2/CYP2B6/CYP3A inducers (US): avoid if possible; if unavoidable, monitor seizure control and adjust fenfluramine according to the local prescribing information. If the inducer is stopped, reduce fenfluramine gradually toward the pre-inducer dose. Strong CYP1A2/2D6 inhibitor present cap at 20 mg/day (no stiripentol) or 17 mg/day(with stiripentol)	[2], [3], [14]
6	Routine monitoring	Track benefit-risk signals continuously.	Weight/BMI, appetite, vitals, daytime alertness; AEs (appetite/weight, GI, somnolence); medication reconciliation at every visit.	[3], [14], [18]
7	Echocardiography schedule	Standardize valve/PASP surveillance; escalate if symptomatic.	ECHO: baseline - > q6 months for 2 years - > annually thereafter. US (REMS): final ECHO 3–6 months post-discontinuation. Bring ECHO forward for new murmur, dyspnea, edema, or exam change.	[2], [3]
8	Benefit assessment (8–16 weeks)	Decide if continuing, optimizing, or pivoting.	Responder thresholds: ≥ 50% and ≥75% reduction; longest inter-seizure interval; rescue-medication use; ER/hospitalizations. Optional brief EF ratings (BRIEF/BRIEF-P) as practice-level signal in DS.	[7], [8], [15], [16], [17], [18], [19], [20]
9	Responder pathway	Consolidate wins; lighten background burden.	Maintain within label; consider simplifying background ASMs to reduce sedation/GI load while preserving control.	[17], [18], [20]
10	Partial responder pathway	Push dose to ceiling and clean DDIs; tailor combos to phenotype.	Optimize to labeled ceiling; address DDIs; consider phenotype-tailored combinations (e.g., predominant GTCs or drop seizures).	[2], [3], [14]
11	Non-responder pathway	Avoid therapeutic inertia.	After optimized titration and DDI cleanup, reconsider regimen; evaluate alternative add-ons/sequencing.	[1], [15], [16], [17], [19], [20]
12	*Re*-evaluation & stopping rules	Protect the heart; taper safely.	Hold/adjust and obtain cardiology input for new/progressive valvular change or clinically significant PASP elevation (per echo lab). Taper gradually on discontinuation to avoid rebound seizures/status.	[2], [3]

**Table 2:** Recommended dosing ceilings and targets (fenfluramine oral solution).

Context	Per-dose target and total daily dose	Daily max / guidance
No stiripentol	0.35 mg/kg twice daily = 0.7 mg/kg/day	26 mg/day
Stiripentol + clobazam regimen⁎	0.2 mg/kg twice daily = 0.4 mg/kg/day	17 mg/day
CYP1A2/2D6 inhibitor present (no stiripentol)	⁎⁎	20 mg/day
CYP1A2/2D6 inhibitor present (with stiripentol)	⁎⁎	17 mg/day
Strong CYP1A2/CYP2B6 inducer present	EU: monitor for reduced efficacy; dose increase may be considered if unavoidable, not exceeding 52 mg/day; reduce gradually when inducer is discontinued. US: avoid strong CYP1A2/CYP2B6/CYP3A inducers where possible; if unavoidable, monitor and follow local maximum-dose guidance.	

⁎ Regimens containing clobazam without stiripentol are managed according to the no stiripentol ceiling (0.35 mg/kg BID; 26 mg/day). Data for fenfluramine with stiripentol in the absence of clobazam are limited; in such off-label scenarios, a conservative approach using the lower ceiling (0.2 mg/kg BID; 17 mg/day) may be considered on an individual basis.

⁎⁎ In the presence of strong/moderate CYP1A2/2D6 inhibitors, no separate weight-based target dose is defined; titrate individually: starting from 0.1 to 0.2 mg/kg/day up to the daily maximum.

The practical contribution of this review is to frame fenfluramine not as a fixed-sequence add-on, but as a monitored, phenotype-guided option whose value depends on seizure profile, tolerability, dose caps, regional interaction guidance, and caregiver-observed functional outcomes.

Looking ahead, priorities include efficacy/safety in adult LGS, confirmatory EF trials with objective endpoints, and comparative efficacy designs that go beyond network meta-analysis and reduce indirectness. Mechanism-based development, whether chiral weighting or sigma-1-selective derivatives, may further optimize the efficacy-tolerability balance. In the meantime, with structured monitoring and disciplined dosing, fenfluramine represents a pragmatic, evidence-based adjunctive treatment for DS and LGS that may be considered earlier in the course of treatment when phenotype and logistics align.

## Declaration of generative AI and AI-assisted technologies in the manuscript preparation process

During the preparation of this work the authors used ChatGPT to improve language and readability. After using this tool, the authors reviewed and edited the content as needed and take full responsibility for the content of the published article.

## CRediT authorship contribution statement

**József Janszky:** Writing – review & editing, Supervision. **Réka Horváth:** Writing – review & editing, Writing – original draft, Conceptualization.

## Ethics statement

Not applicable. This is a narrative review and did not involve new studies of human participants or animals.

## Funding

This article was supported by the 10.13039/501100012550National Research, Development and Innovation Fund of Hungary (NKFIH-K142479) and by the National Laboratory of Translational Neuroscience within the “Adult Nervous System Disorders” (RRF-2.3.1-21-2022-00011) program framework.

## Declaration of competing interest

The authors declare that they have no known competing financial interests or personal relationships that could have appeared to influence the work reported in this paper.

## Data Availability

Data sharing is not applicable to this article as no new data were created or analyzed.

## References

[1] Guerrini R., Chiron C., Vandame D., Linley W., Toward T. (2024). Comparative efficacy and safety of first-line add-on therapies for seizures in Dravet syndrome: a network meta-analysis. Epilepsia Open.

[2] European Medicines Agency (EMA) (2025). Fintepla: EPAR - product information. https://www.ema.europa.eu/en/medicines/human/EPAR/fintepla.

[3] U.S. Food and Drug Administration (2025). FINTEPLA (fenfluramine) oral solution: prescribing information. https://dailymed.nlm.nih.gov/dailymed/drugInfo.cfm?setid=e88f360e-33ad-4cd6-b2de-5ef885857c5d.

[4] Sourbron J., Lagae L. (2023). Fenfluramine: a plethora of mechanisms?. Front Pharmacol.

[5] Martin P., de Witte P.A.M., Maurice T. (2020). Fenfluramine acts as a positive modulator of sigma-1 receptors. Epilepsy Behav.

[6] Martin P., Maurice T., Gammaitoni A.R. (2021). An emerging role for sigma-1 receptors in developmental and epileptic encephalopathies; fenfluramine as a case example. Int J Mol Sci.

[7] Bishop K.I., Isquith P.K., Gioia G.A. (2021). Improved everyday executive functioning after profound seizure reduction with fenfluramine: Dravet syndrome phase 3 open-label extension analysis. Epilepsy Behav.

[8] Bishop K.I., Isquith P.K., Gioia G.A. (2023). Fenfluramine and executive function in preschool Dravet syndrome (<5 years): a critical period. Epilepsy Behav.

[9] Dinday M.T., Baraban S.C. (2015). Large-scale phenotype-based antiepileptic drug screening in a zebrafish model of Dravet syndrome. eNeuro.

[10] Sourbron J., Braat S., al Zaabi S. (2016). Serotonergic modulation as an effective treatment in SCN1A mutant zebrafish. ACS Chem Neurosci.

[11] Sourbron J., Smolders I., De Witte P., Lagae L. (2017). Pharmacological analysis of fenfluramine mechanisms in SCN1A mutant zebrafish. Front Pharmacol.

[12] Li J., Nelis M., Sourbron J. (2021). Efficacy of fenfluramine and norfenfluramine enantiomers in Dravet zebrafish. Neurochem Res.

[13] Erenburg N., Perucca E., Bechard J. (2024). Stereoselective analysis of the antiseizure activity of fenfluramine and norfenfluramine in mice. Int J Mol Sci.

[14] Martin P., Czerwinski M., Limaye P.B. (2022). In vitro evaluation of fenfluramine and norfenfluramine as victims of drug interactions. Pharmacol Res Perspect.

[15] Lagae L., Sullivan J., Knupp K. (2019). Fenfluramine hydrochloride for the treatment of seizures in Dravet syndrome: a randomized, double-blind, placebo-controlled trial. Lancet.

[16] Nabbout R., Mistry A., Zuberi S.M. (2020). Fenfluramine for treatment-resistant seizures in patients with Dravet syndrome receiving stiripentol-inclusive regimens: a randomized clinical trial. JAMA Neurol.

[17] Sullivan J., Scheffer I.E., Lagae L. (2020). Fenfluramine HCl provides long-term clinically meaningful reduction in seizure frequency: open-label extension analysis. Epilepsia.

[18] Boncristiano A., Balestrini S., Doccini V. (2025). Fenfluramine treatment for Dravet syndrome: long-term real-world analysis demonstrates safety and reduced healthcare burden. Epilepsia.

[19] Knupp K.G., Scheffer I.E., Ceulemans B. (2022). Efficacy and safety of fenfluramine for the treatment of seizures associated with Lennox-Gastaut syndrome: a randomized clinical trial. JAMA Neurol.

[20] Knupp K.G., Scheffer I.E., Ceulemans B. (2023). Fenfluramine provides clinically meaningful reduction in drop seizures in Lennox-Gastaut syndrome: open-label extension interim analysis. Epilepsia.

[21] Knupp K.G., Scheffer I.E., Schoonjans A.S., Sullivan J., Lagae L., Auvin S. (2025). Final analysis from an open-label extension study of fenfluramine for the treatment of seizures in Lennox-Gastaut syndrome: long-term impact on patients and caregivers. Epilepsy Behav.

[22] Devinsky O., King L., Schwartz D., Conway E., Price D. (2021). Effect of fenfluramine on convulsive seizures in CDKL5 deficiency disorder. Epilepsia Open.

[23] Specchio N., Marsh E., Devinsky O. (2025). AES annual meeting 2025; Abstract 2.429.

[24] Geenen K.R., Doshi S.P., Patel S. (2021). Fenfluramine for seizures in Sunflower syndrome. Dev Med Child Neurol.

[25] Bishop K.I., Isquith P.K., Gioia G.A., Knupp K.G., Scheffer I.E., Sullivan J. (2021). Fenfluramine treatment improves everyday executive functioning in patients with Lennox-Gastaut syndrome: analysis from a phase 3 clinical trial. Neuropediatrics.

[26] Bishop K.I., Isquith P.K., Roth R.M. (2022). AES annual meeting.

[27] Breuillard D., Knupp K., Strzelczyk A. (2025). AES annual meeting 2025.

[28] Abenhaim L., Moride Y., Brenot F. (1996). Appetite-suppressant drugs and the risk of primary pulmonary hypertension. N Engl J Med.

[29] Connolly H.M., Crary J.L., McGoon M.D. (1997). Valvular heart disease associated with fenfluramine-phentermine. N Engl J Med.

[30] Mark E.J., Patalas E.D., Chang H.T., Evans R.J., Kessler S.C. (1997). Fatal pulmonary hypertension associated with short-term use of fenfluramine and phentermine. N Engl J Med.

[31] Lattanzi S., Trinka E., Russo E. (2023). Pharmacotherapy for Dravet syndrome: a systematic review and network meta-analysis of randomized controlled trials. Drugs.

